# Changes in Porcine Corpus Luteum Proteome Associated with Development, Maintenance, Regression, and Rescue during Estrous Cycle and Early Pregnancy

**DOI:** 10.3390/ijms222111740

**Published:** 2021-10-29

**Authors:** Pawel Likszo, Dariusz Jan Skarzynski, Beenu Moza Jalali

**Affiliations:** Institute of Animal Reproduction and Food Research, Polish Academy of Sciences, Tuwima 10, 10-748 Olsztyn, Poland; p.likszo@pan.olsztyn.pl (P.L.); d.skarzynski@pan.olsztyn.pl (D.J.S.)

**Keywords:** corpus luteum, porcine, proteomics, steroidogenesis, luteal regression

## Abstract

Corpus luteum (CL), a transitory gland, undergoes rapid growth in a limited time to produce progesterone (P4) followed by its regression. A complex molecular signaling is involved in controlling luteal P4 production. In the present study, 2D gel electrophoresis-based proteomics and in silico functional analysis were used to identify changes in key proteins and pathways in CL along the different stages of the estrous cycle as its development progresses from early (Day 3) to mid-luteal phase (Day 9), effective functioning (Day 12) followed by regression (Day 15) or, in the case of pregnancy, rescue of function (Day 15). A total of 273 proteins were identified by MALDI-MS/MS analysis that showed significant changes in abundances at different stages of CL development or regression and rescue. Functional annotation of differentially abundant proteins suggested enrichment of several important pathways and functions during CL development and function maintenance including cell survival, endocytosis, oxidative stress response, estradiol metabolism, and angiogenesis. On the other hand, differentially abundant proteins during CL regression were associated with decreased steroid synthesis and metabolism and increased apoptosis, necrosis, and infiltration of immune cells. Establishment of pregnancy rescues CL from regression by maintaining the expression of proteins that support steroidogenesis as pathways such as the super-pathway of cholesterol biosynthesis, RhoA signaling, and functions such as fatty acid metabolism and sterol transport were enriched in CL of pregnancy. In this study, some novel proteins were identified along CL development that advances our understanding of CL survival and steroidogenesis.

## 1. Introduction

One of the factors that determine pregnancy success in mammals is production of optimal quantities of progesterone (P4) by corpus luteum (CL). In pigs, once the CL is formed from the ruptured follicle after ovulation, it starts secreting progesterone (P4), which is required for the establishment and maintenance of pregnancy. The P4 concentrations reach the peak values during the mid-luteal phase, between the Days 9–12 of the estrous cycle, coinciding with maternal recognition of the pregnancy period. In the absence of timely appropriate embryonic signals, plasma P4 concentrations start to decrease, leading to functional luteolysis followed by structural luteolysis, which gives rise to initiation of a new reproductive cycle [1]. Rescue of the CL function to maintain P4 production is imperative for a successful pregnancy establishment. Actions of P4 not only lead to development of endometrial receptivity for the embryo implantation but also support the growth of the embryo [2]. Besides endocrine factors, many proteins are produced locally in the CL to maintain CL function. Dysregulation of the luteal protein components may have adverse effects on luteal function and also on the endometrial function, leading to embryo loss or implantation failure [3]. Luteal dysfunction, arising due to abnormal steroidogenesis, leads to early embryonic loss and pregnancy failure in livestock [4], humans [3,5], ruminants [6], and pigs [7]. The CL is the sole source of P4 throughout the gestation period in pigs. Thus, the underlying molecular mechanisms controlling P4 production during the development, function maintenance, and rescue of the CL is of paramount importance to understanding a porcine fertile cycle.

Deciphering mechanisms underlying the process of steroidogenesis and regression of CL in many species has been the subject of considerable research [2]. Using targeted analysis approach, tremendous work has been done on understanding molecular regulation of CL biology. Several mechanisms and factors regulating luteal growth and regression are known that involve lipid metabolism in CL [8,9], angiogenesis [10,11] and apoptosis [12,13,14], extracellular matrix remodeling [15], immune-endocrine cross-talk [16,17], role of tumor necrosis factor alpha and macrophages [18,19,20], and enzymes involved in steroidogenesis [1]. The CL during the estrous cycle undergoes tremendous changes in its structure and function. These changes are driven by a balance between principal luteotrophic and luteolytic signals. In the absence of embryonic signals, under the influence of endometrial prostaglandin (PG)F2α, a luteolytic factor in mammalian species including pigs, CL undergoes regression. In pigs, however, endogenous PGF2α is luteolytic only after Day 12 of the estrous cycle. Molecular control of luteolytic sensitivity to endogenous PGF2α has not been properly understood; however, a role for PGF2α-induced estradiol biosynthesis has been suggested [21]. During pregnancy, an abundance of luteal estradiol and PGE2 are reported to redirect PGF2α secretion from the endocrine to exocrine direction [22] and regulate specific pathways responsible for steroidogenesis and angiogenesis that rescues CL from luteolysis in pigs [10]. In spite of much information on the molecular control of porcine CL function, regression, and rescue, an increased understanding of factors regulating CL development, maintenance of function, regression, and rescue from regression is still lacking and is critical to improvement in reproductive efficiency.

The application of proteomics to understand luteal development and regression can yield new insights into the understanding of physiological and biological processes associated with CL. There has been only one study reporting proteomic changes associated with CL formation [23], no study deciphering porcine luteal proteome, and very few reports of proteomic analysis of CL function in other species such as ewe, bovine, and rat [24,25,26]. These studies on ovine and rat CL found differentially expressed proteins involved in signal transduction, oxidative stress, and metabolism. Though the protein categories that changed in abundance during different stages of luteal cycle were the same, very few proteins belonging to these categories were common across the three species studied. This necessitates the investigation of CL from different species, particularly when the physiology of a process such as CL development and regression, as in the case of pigs, differs considerably. Using proteomics, we identified sequential changes in protein expression from its development from an early luteal phase to an early mid-luteal phase (Days 3–9), functional maintenance during mid-luteal phase (Days 9–12), and to regression (Days 12–15) inclusive of changes that are responsible for luteal rescue (Day 15 of cycle vs. Day 15 of pregnancy). This study identified novel proteins, not reported in porcine CL before, and provides a source for exploring the functional roles of proteins associated with important luteal functions.

## 2. Results

### 2.1. Progesterone Concentrations

Plasma progesterone (P4) concentrations reflected the growth, regression, and rescue of CL during the estrous cycle and pregnancy. P4 concentrations increased exponentially from Day 3 to Day 9 of the estrous cycle (*p* < 0.01) and remained high until Day 12 of the estrous cycle. A significant decrease in P4 was observed on Day 15 as compared to Day 12 of the estrous cycle (*p* < 0.01). The plasma P4 concentrations on Day 15 of pregnancy were similar to those observed on Days 9–12 of the estrous cycle (Table S1).

### 2.2. Proteomic Changes Associated with Growth and Development of CL on Days 3, 9, and 12 of the Estrous Cycle

**Figure 1:**
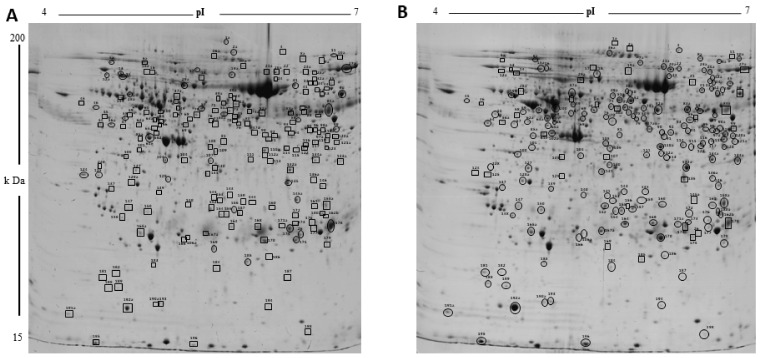
The two-dimensional gel electrophoresis analysis of corpus luteum proteins. Representative 2DE gel image of differentially abundant protein spots in corpus luteum on (**A**) Day 3 and (**B**) Day 9 of the estrous cycle. There were 191 spots that changed significantly (*p* < 0.05) between two the groups, and are shown in the map and indicated by numbers. Circles and squares designate spots with increase and decrease in abundances, respectively on a day of estrous cycle. Proteins corresponding to spot numbers are listed in Table S2.

To analyze CL proteins that differ in their abundances with growth and development, a 2D-PAGE of porcine luteal protein lysates from Days 3, 9, and 12 of the estrous cycle was performed using IEF in the pH range of 4–7 followed by PAGE on large-format SDS gels (12.5%). ImageMaster 2D Platinum Software was used to compare the 2D gels from Day 3 to Day 9 and Day 9 to Day 12 of the estrous cycle (DC). Protein spots showing statistically significant differences in normalized spot volumes with development and growth of CL from Day 3 to Day 9 (Figure 1) and between Day 9 and Day 12 of the estrous cycle were marked (Figure S1). The comparison of luteal proteome between Day 3 and Day 9 revealed that, whereas 154 protein spots were more abundant on Day 9, 37 spots were more abundant on Day 3. Many protein spots (37) were observed only on Day 9 of the estrous cycle when compared to Day 3. Moreover, between Day 9 and Day 12, whereas 56 proteins were more abundant on Day 12, only 11 proteins were expressed at higher levels on Day 9. The differentially abundant protein spots found in at least three gels were picked for identification. A list of all the identified proteins between different comparisons is provided in Supplementary Tables S2 and S3. The identified proteins that were more abundant on Day 3 included serine protease inhibitors (SERPINC1, SERPIN-A5, SERPINA3-6, and SERPINA3-8) and complement system (C1S, C3, C4A, and CFB). More abundant proteins on Day 9 of the estrous cycle included transport protein (CLTA, APOA1, FABP3, and SELENBP1), protection against oxidative stress (PRDX4, PRDX6, and SLC25A24), chaperone protein (HSPA5, DNAJB11, ERP29, HSPB1, HSPB6, HSP90AA1, FKBP5, ERP44, and CCT5), and proteins regulating cell motility (ACTB, ACTR3, CLIC5, and CAPG).

**Figure 2:**
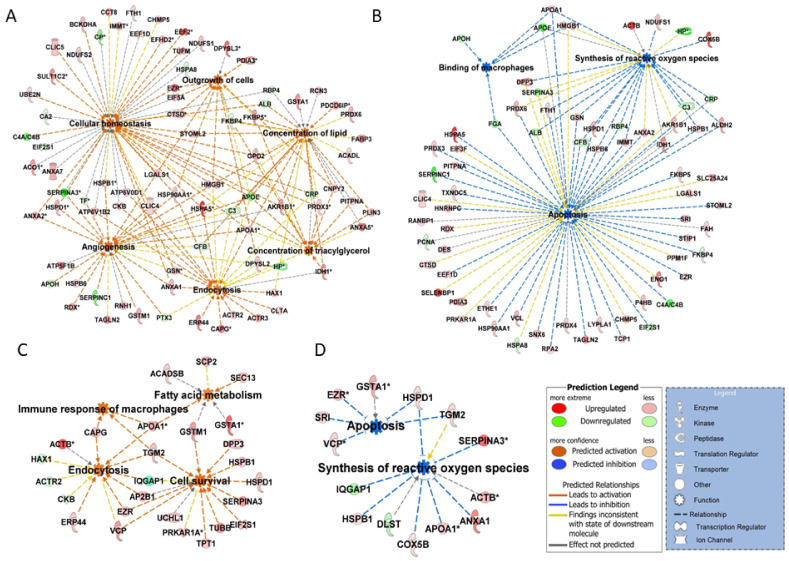
IPA disease and function analysis of differentially abundant proteins. Top functions ranked in accordance to z-score (−2.0 ≥ z-score ≥ 2.0) with luteal development from Day 3 to Day 9 of estrous showed (**A**) enrichment of functions: cellular homeostasis, concentration of lipids and triacylglycerol, endocytosis and angiogenesis, and (**B**) inhibition of functions: apoptosis, synthesis of reactive oxygen species, and binding of macrophages. Maintenance of luteal function from Day 9 to Day 12 showed (**C**) enrichment of functions: fatty acid metabolism, cell survival, endocytosis, and immune response of macrophages and (**D**) inhibition of functions: apoptosis and synthesis of reactive oxygen species. Red and green colors depict an increase or decrease, respectively, in abundance of the proteins associated with a comparison.

Functional annotation of differentially abundant proteins using IPA analysis linked these proteins to top canonical pathways that included negative acute phase response, xenobiotic metabolism, LXR-RXR-signaling, RhoA signaling, and clathrin-mediated endocytosis signaling (Table 1). The disease and function analysis clearly revealed that the processes that were enriched during the mid-luteal phase on Day 9 support luteal growth and steroidogenesis. Proteins associated with functions such as cellular growth, endocytosis, angiogenesis, and concentration of lipids and triacylglycerol were significantly more abundant in Day 9 of corpus luteum (Figure 2A). Moreover, proteins that inhibit apoptosis and counter free radicals generated were also enriched with CL development (Figure 2B).

Maintenance of the CL function (Day 9 to Day 12) was associated with a further increase in protein abundances of the enzymes of steroid biosynthesis pathway (HMGCS1 and APOA1), cell survival (EIF2S, HSPD1, HSPB1, UCHL1, DPP3), and proteins associated with cell migration (DYNC1I2, VIM, TUBB, TPT1, IQGAP1, MAPRE1, and CAPG). Some of the proteins associated with alleviating oxidation stress continued to be present at increased abundances on Day 12 of the estrous cycle. Additionally, a pathway that was enriched on Day 12 had an immune response to macrophages (Figure 2C,D). Some of the proteins started to show a decreasing trend towards Day 12 of estrous cycle, most prominent of those were associated with energy homeostasis (CKB), oxidative stress (GSS), and transport proteins such as GC. Supplementary Figure S2 shows changing trends in the spot volumes of proteins associated with steroidogenesis and oxidation stress response.

### 2.3. Proteomic Changes Associated with Regression of CL on Day 15 of the Estrous Cycle

**Figure 3:**
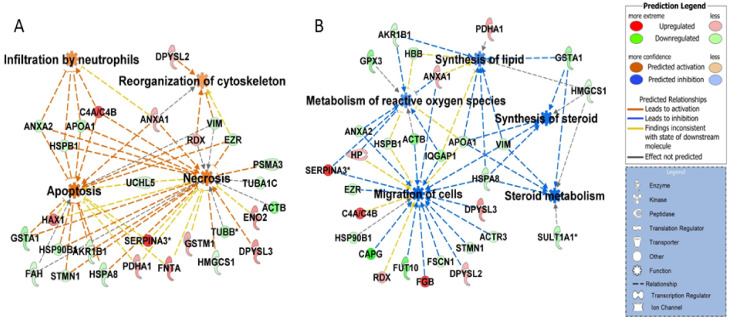
IPA disease and function analysis of differentially abundant proteins. Top functions ranked in accordance to z-score (−2.0 ≥ z-score ≥ 2.0) with corpus luteum regression on Day 15 as compared to Day 12 of the estrous cycle showed (**A**) enrichment of functions: apoptosis, necrosis, reorganization of cytoskeleton, and infiltration by neutrophils and (**B**) inhibition of functions: synthesis of lipids, synthesis of steroid, steroid metabolism, metabolism of reactive oxygen species, and migration of cells. Red and green colors depict an increase or decrease, respectively, in abundance of the proteins.

The molecular changes associated with luteal regression were identified by a comparison between 2DE gels obtained using luteal protein lysates from Day 12 (functional) and Day 15 (regression) of estrous cycle. A total of 43 proteins were more abundant in Day 12 of CL as compared to 32 proteins that were more abundant on Day 15 of the estrous cycle. A spot map of differentially abundant proteins is presented in Supplementary Figure S3 and a list of all the identified proteins is given in Supplementary Table S4. Proteins associated with cell organization (ACTR3, DYNC1I2, EZR, FSCN1, CAPG, IQGAP1, TUBA1C, TUBB, STMN1, VIM), translation process (FUT10, HNRNPH1), lipid metabolism (APOA1, FDPS, HMGCS1), chaperone activity (HSP90B1, HSPB1, HSPA8, PSMA3, PDIA6), and oxidative stress response (GPRX, GSTA1, GSTM2) were more abundant on Day 12 of CL. On the other hand, proteins associated with cell motility/remodeling (DPYSL2, DPYSL3, HAX1, PDX), acute phase response (FGB, C4A, CKB), and proteases (CLPP, SH3GLB2, HYAL1) were more abundant in CL on Day 15 of estrous cycle. The functional annotation of differentially abundant proteins revealed that unfolded protein response, oxidative stress response pathways, and synthesis of steroid were inhibited on Day 15 and there was an increase in abundance of proteins associated with apoptosis, infiltration of neutrophils, and cytoskeletal reorganization (Figure 3A,B).

### 2.4. Proteomic Changes Associated with Rescue of CL Function on Day 15 of Pregnancy

In pigs, recognition of the pregnancy signal is conceptus-secreted estradiol (E2) between Days 11–13 of pregnancy, which leads to rescue of luteal function in terms of continuation of P4 production by CL. To evaluate the molecular changes that deviate CL towards function rather than regression during pregnancy, we compared 2D gels from Day 15 of the estrous cycle with that of Day 15 of pregnancy. Whereas a total of 67 luteal proteins showed increased abundances on Day 15 of pregnancy, 24 proteins were more abundant on Day 15 of the estrous cycle (Figure S4 and Table S5). An expected significant increase in enzymes involved in steroidogenesis was observed on Day 15 of pregnancy as the abundance of HSD3B1, G6PD, GSTA1, IDI1, STNM1, and HMGCS1 was significantly increased. Moreover, an increase in the expression of anti-apoptotic proteins was also observed in CL of Day 15 of pregnancy. The other class of proteins that was more abundant included COX5B, CP, G6PD, and GSTA.

**Figure 4:**
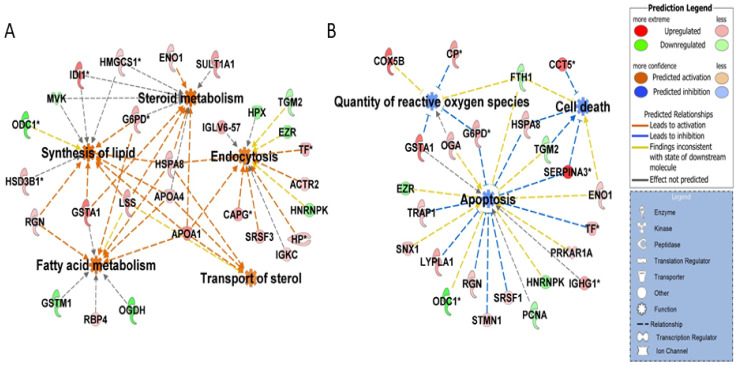
IPA disease and function analysis of differentially abundant proteins: Top functions ranked in accordance to z-score (−2.0 ≥ z-score ≥ 2.0) with rescue of corpus luteum function on Day 15 of pregnancy as compared to Day 15 of the estrous cycle showed (**A**) enrichment of functions: synthesis of lipid, endocytosis, transport of sterol, steroid and fatty acid metabolism and (**B**) inhibition of functions: apoptosis, cell death, and quantity of reactive oxygen species. Red and green colors depict an increase or decrease, respectively, in abundance of the proteins.

Functional analysis revealed clathrin-mediated endocytosis and super-pathway of cholesterol biosynthesis to be one of the topmost enriched canonical pathways in CL of pregnancy (Table 1). Proteins associated with lipid metabolism and cell survival were also more abundant on Day 15 of pregnancy (Figure 4A) and a luteal rescue was associated with a significant decrease in proteins associated with apoptotic signaling, cell death signaling, and generation of reactive oxygen species (Figure 4B).

### 2.5. ToppCluster Analysis

**Figure 5:**
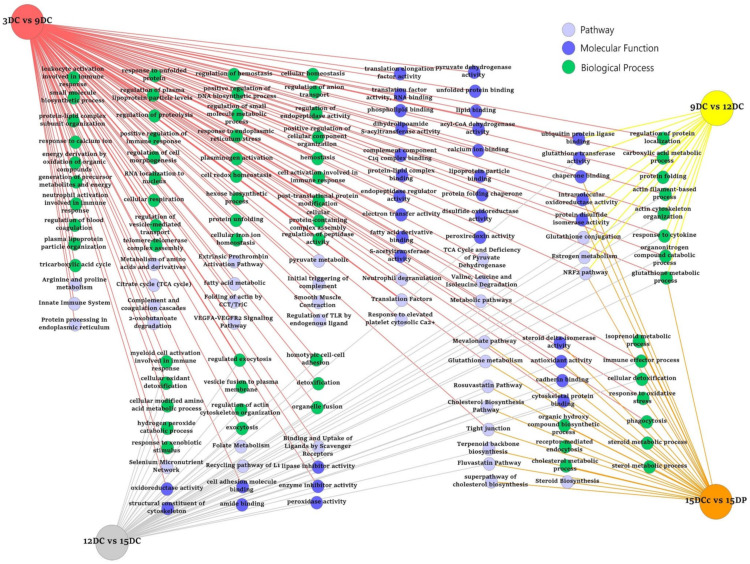
ToppCluster tool was used to analyze differentially abundant proteins associated with molecular function (dark blue), biological process (green), and pathways (light blue). The functional map illustrates shared and group-specific functional annotation terms generated in multicluster protein functional enrichment analysis for differentially abundant proteins identified in porcine corpus luteum in various comparisons: 3DC vs. 9DC (red circle), 9DC vs. 12DC (yellow circle), 12DC vs. 15DC (gray circle), and 15DC vs. 15DP (orange circle). The design of this network was modified in Cytoscape. The complete results are presented in a tabular format in Supplementary Table S6.

A further analysis of the biologic categories that change significantly during the lifespan of the corpus luteum, pathway, and GO terms (Biological Process, Molecular Function) was performed using ToppCluster tool. We compared differentially abundant proteins identified in the CL samples collected on different days of the estrous cycle and luteal rescue during pregnancy. This analysis resulted in a network representation of shared and luteal phase-specific pathways and GO terms for growth, maintenance, regression, or rescue of CL generated by comparing multiple protein lists (Figure 5). Redundant and non-informative terms were manually removed for a clear presentation. Complete ToppCluster results are reported in Table S6. Out of 35 enriched pathways and functions obtained in the ToppCluster tool, 21 pathways and functions were specifically enriched in mid-luteal CL on Day 9 that included protein processing in endoplasmic reticulum, plasma lipoprotein particle organization, fatty acid metabolism, amino acid metabolism, VEGF-VEGFR2 signaling, response to calcium ion, and branched chain amino acid degradation. On Day 12, the pathway that was specific to comparison 9 vs. 12 was the estrogen metabolism pathway. On Day 15, infiltration by neutrophils and cellular infiltration were the enriched pathways. The CL of pregnancy showed specific enrichment of a superpathway of cholesterol biosynthesis. All luteal phases investigated revealed shared pathways and functions that included cellular response to cytokine, actin cytoskeleton organization, NRF2-mediated oxidation stress response, and carboxylic acid metabolism process.

### 2.6. Validation of 2DE Results

**Figure 6:**
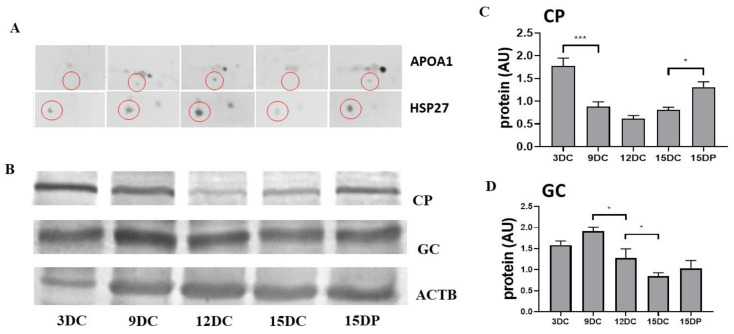
Western blot validation of change in abundances of proteins: (**A**) Abundance of proteins (red circles)APOA1 and HSP27 by 2D WB and (**B**) CP and GC by 1D WB in corpus luteum, selected for validation of 2DE. The statistical analysis of the protein abundance analysis of four biological repeats of (**C**) CP and (**D**) GC relative to the loading control (ACTB). Data were analyzed using one-way ANOVA with Sidak multiple comparison tests and are presented as mean ± SEM. AU—arbitrary units. * and *** indicate *p* < 0.05 and *p* < 0.001.

To validate and verify the expression of proteins identified by mass spectrometry after 2DE analysis, we performed Western blot (WB) analysis in a one/two-dimensional gel system. Proteins that appeared in all the investigated categories were selected for validation. Corresponding to 2DE results, WB analysis showed a significantly low abundance of APOA1 and HSP27 on Days 3 and 15 of the estrous cycle, compared with Days 9 and 12 of the estrous cycle and Day 15 of pregnancy in CL (Figure 6A). Abundance of ceruloplasmin (CP), acute phase response protein, was highest in early CL on Day 3 followed by a decrease on Days 9, 12, and 15 of the estrous cycle. Rescue of luteal function on Day 15 of pregnancy was associated with an increased abundance of CP (Figure 6B,C). A significant decrease in protein abundance of vitamin D binding protein (GC) was observed in the mid-luteal phase on Day 12 and during regression on Day 15 of the estrous cycle as compared to Day 9. However, contrary to 2DE results, WB analysis did not reveal any significant difference in GC between Day 15 of cycle and pregnancy (Figure 6D).

**Figure 7:**
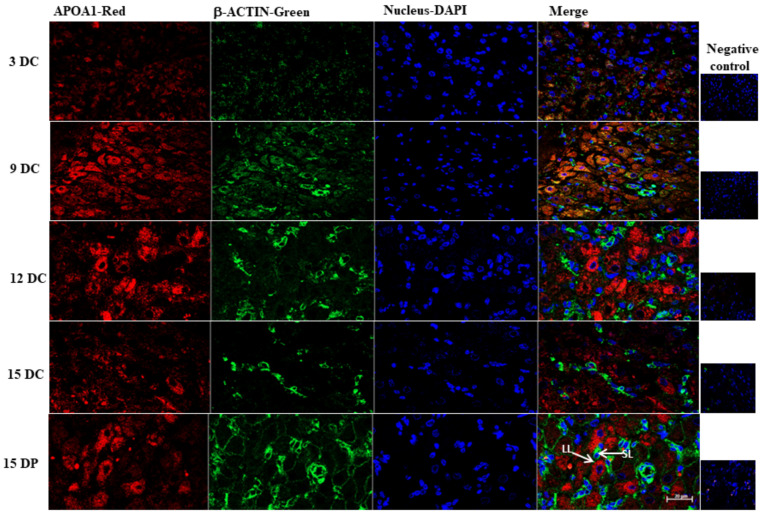
Localization of sterol transport protein in porcine corpus luteum. Representative confocal microscope images of immunofluorescent images of APOA on various days of the estrous cycle (DC) and Day 15 of pregnancy (DP). Maximum immunoreactivity of APOA1 was observed on 12 DC and 15 DP. LL—large luteal cells, SL—small luteal cells. Scale bar represents 20 μm.

To further validate the differentially expressed protein APOA1 that showed variations in abundance throughout the luteal phases investigated and evaluate its localization in the corpus luteum, we performed immunofluorescence analysis. Immunofluorescence analysis showed APOA1 was mainly localized in the large luteal cells (Figure 7). Immunoreactivity of APOA1 was stronger on Days 9 and 12 of the estrous cycle and Day 15 of pregnancy compared to Day 3 and 15 of the estrous cycle.

### 2.7. Effect of Triclosan on P4 Secretion by Luteal Explants

**Figure 8:**
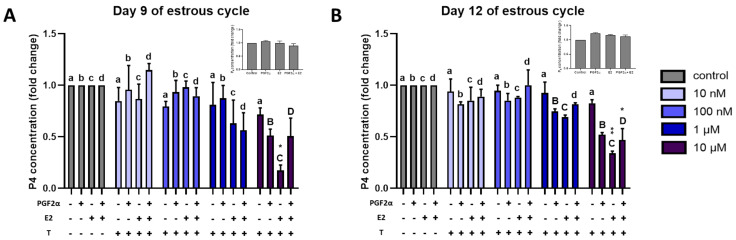
Progesterone concentrations in the corpus luteum explant-conditioned medium. The effect of triclosan (10 nM, 100 nM, 1 µM, and 10 µM) in combination with PGF2α (1 µM), E2 (10 nM), and PGF2α + E2 on progesterone secretion in the culture medium by a luteal explant collected on (**A**) Day 9 of the estrous cycle and (**B**) Day 12 of the estrous cycle. Insets in the Figure show no significant effect of PGF2α, E2, and PGF2α + E2 on progesterone secretion by luteal explant collected on Days 9 and 12 of the estrous cycle. Data are presented as mean ± SEM and were analyzed by one-way ANOVA with Sidak’s multiple comparison test. Small and capital letters denote a statistically significant difference between the comparison. Asterisks denote significant differences between T-only-treated explants and T in combination with E2 or PGF2α + E2 (* *p* < 0.05 and ** *p* < 0.01).

Proteomic results of the present study revealed a differential regulation of an estrogens-metabolizing enzyme with luteal development, regression, and rescue. To gain an understanding of the possible role of estrogen sulfotransferases in the porcine luteal function, and their possible role in rendering CL sensitive to PGF2α actions, luteal explants collected on Days 9 and 12 of the estrous cycle were treated with PGF2α, E2, sulfotransferase inhibitor, Triclosan (T), and a combination of these factors. Triclosan concentrations used in the experiments did not induce tissue damage in CL explants as measured by LDH activity in culture medium after 24 h of incubation. The effect of these treatments on in vitro P4 production by luteal explants was analyzed (Figure 8). Concentration of P4 after in vitro stimulation with 1 μM PGF2α, 10 nM E2, and PGF2α + E2 did not show any significant effect on P4 production (insets in Figure 8). Furthermore, treatment of CL explants with PGF2α or E2 in the presence of lower concentrations of T (10 nm and 100 nM) did not induce any change in P4 concentrations. However, T at 10 µM in combination with PGF2α, E2, and PGF2α + E2 significantly decreased luteal P4 secretion from explants compared to respective control (PGF2α-, E2-, and PGF2α + E2-treated) CL explants on Days 12 and 9 of the estrous cycle. We observed the lowest P4 concentration in a medium with E2 + T treatment on both days tested. However, on Day 12, as low as 1 μM Triclosan was sufficient to inhibit P4 secretion from explants treated with PGF2α and E2 (Figure 8).

## 3. Discussion

In the present study, 273 proteins were identified that showed a change in their abundances depending upon the different stages of the luteal life span. Some of the proteins in our data set were not reported in porcine CL before. Significant changes in the most number of proteins were observed during development of CL from the early luteal phase (Day 3) to the mid-luteal phase (Day 9). Using bioinformatics tools for interpretation of molecular changes, a deeper insight into functions of these proteins was gained and key pathways responsible for regulating CL function were identified. Some of the proteins were associated with many pathways and only a few specific proteins belonged to a particular group only. We presented comprehensive information on (1) the changes in the luteal proteome during development and regression of CL and (2) the changes in protein expression associated with rescue of the luteal function during pregnancy. Top pathways and biological function categories that were regulated with luteal development and function involved lipid and carbohydrate metabolism, oxidative stress response, angiogenesis, receptor-mediated endocytosis, LXR/RXR activation, and pathways such as RhoA signaling and estrogen receptor signaling. On the other hand, pathways such as infiltration of neutrophils, apoptosis, and necrosis were enriched during regression along with altered cellular migration and steroid metabolism.

### 3.1. Steroid Biosynthesis

To produce high levels of progesterone to support a possible pregnancy, many pathways supporting steroid biosynthesis and metabolism and sterol transport were overrepresented in functional CL during the mid-estrous cycle and in CL of pregnancy. It has been suggested that the main source of cholesterol for luteal P4 synthesis is endocytosis of cholesterol-rich, low-density lipoproteins, as in pigs, or selective uptake of cholesterol ester from-high density lipoproteins, as in ruminants [27]. Recently, proteins involved in de novo cholesterol synthesis have also been identified in the porcine CL [23]. Our results suggest that multiple mechanisms are active in functional CL to produce P4. We observed that proteins associated with receptor-mediated endocytosis such as clathrin light chain (CLTA), actin-related proteins (ACTR2, ACTR3), capping protein (CAPG), sorting nexin 6 (SNX6), HSPA8, and apolipoproteins were overrepresented in functional CL during the estrous cycle and early pregnancy with a significant decrease in their abundance during luteolysis. Steroidogenic cells intake LDL in an endocytic mechanism where apolipoproteins such as APOE or APOC are internalized in clathrin-coated pits and degraded, releasing free cholesterol for steroid biosynthesis. Whereas the role of actin dynamics in receptor-mediated endocytosis in yeast is well established, in mammalian cells it is still controversial. However, some studies, using total internal reflection fluorescence, have demonstrated control of actin dynamics through actin-related proteins on some subsets of the clathrin-coated pits in mouse fibroblasts [28]. Moreover, similarities in actin dynamics and the role of ACTR2/ACTR3 and other proteins such as dynamin and sorting nexins between yeast and mammalian clathrin-mediated endocytosis have also been shown [29,30]. In regressing CL, as proteins associated with endocytic machinery were decreased significantly, our study supports the previous results suggesting that regressing CL loses its ability to utilize LDL as a source of cholesterol [31]. Endocytosis for steroid biosynthesis in CL is known, but involvement of specific proteins of this pathway were not reported in the porcine CL before.

We observed an increased abundance of proteins involved in the cholesterol biosynthesis including sterol transport from plasma in the mid-luteal phase CL and in CL of pregnancy. An increase in APOA1, vitamin D binding protein (GC), and SCP2 was specific for the mid-luteal phase. APOA4 and APOR, on the other hand, were detected at higher levels only in the CL of pregnancy. APOA1, a major component of HDL, was previously reported to be highly expressed in the mid-luteal phase and during early pregnancy in ovine and bovine CL, where it was suggested to induce production of P4 and luteotrophic PGE2 [24,32]. SCP2, a lipid transfer protein that plays a key role in the pre- and transmitochondrial movement of cholesterol [33], was highly abundant in the mid-luteal phase. In rats, SCP2 is expressed in luteal cells during pregnancy [34] and PGF2α is reported to reduce cholesterol transport by decreasing SCP2 expression [35]. Proteins involved in the cholesterol synthesis pathway were significantly increased in functional CL of the estrous cycle and in CL of pregnancy. Different enzymes in cholesterol biosynthesis pathway showed increased abundances in different luteal phases and during early pregnancy. Enzymes such as HMGCS1 and IDI1 were increased during the mid-luteal phase and early pregnancy and FDPS was detected only in the mid-luteal phase. We also observed a pregnancy-specific increase in the abundance of enzyme LSS. Moreover, many of the enzymes such as large, medium, and small chain acyl co-A, which catalyze the initial step of β-oxidation of fatty acids, were present in higher abundances in the early mid-luteal phase CL and some of these enzymes showed an increase also in regressing CL. Biosynthesis of cholesterol is increased by fatty acid metabolism and there is a link between catabolism of branched chains’ amino acids and cholesterol biosynthesis [36]. In porcine CL, enzymes catabolizing branched chain amino acids such as BCKDHB, BCKDHA, and HIBCH were significantly more abundant in the mid-luteal phase. Another mechanism that can contribute towards cholesterol synthesis is the accumulation of triacylglycerols. Triacylglycerols are stored as cytoplasmic ‘lipid droplets’ enclosed by a monolayer of phospholipids and hydrophobic proteins such as the perilipins (PLIN). An enrichment of the proteins that increase triacylglycerol concentration including PLIN3 and PITPNA was observed in the early mid-luteal phase. The importance of lipid droplets in bovine luteal function and steroidogenesis was recently reported [37]. These results suggest that, to sustain optimal P4 production, intake of lipids by endocytosis and de novo cholesterol synthesis plays a major role in functional CL.

Additionally, cholesterol biosynthesis, in our study, abundance of HSD3B1, a key protein in P4 biosynthesis, was significantly higher in the mid-luteal phase and during pregnancy as compared to early-luteal CL. Our results suggest that all the components of P4 production are elevated during the mid-luteal phase followed by a decrease in proteins associated with the cholesterol biosynthesis pathway.

### 3.2. RhoA Signaling

Cytoskeleton-associated proteins are emerging as novel regulators of luteal function. Ras homolog gene family member A (RhoA) signaling was altered during different phases of the estrous cycle. In CL, RhoA regulates the dynamics of cytoskeleton (37). Proteins acting both upstream and downstream of RhoA such as EZR and RDX along with other cytoskeletal proteins, ACTB, ACTR2, ACTR3, VIM, MYL2, MYL6, and SEPTIN2, were highly abundant during the early and mid-luteal phases with a decrease in their abundance during luteolysis. The importance of microfilaments and microtubules in LH-induced P4 synthesis was shown in porcine luteal cells [38]. Moreover, vimentin-negative mice (Vim-/-) showed a significant decrease in P4 production [39]. The cytoskeleton regulated by actin filaments and vimentin might transport cytoplasmic cholesterol to mitochondria [40,41]. Though the role of cytoskeleton-associated proteins in ovarian steroidogenesis is not very well investigated, their importance in adrenal steroidogenesis is well established [41,42]. The study by El Zowalaty points towards a role of cytoskeletal proteins in lipid transport and regulation of mitochondrial density, which might affect progesterone synthesis [43].

### 3.3. Estrogen Metabolism

Enzymes involved in xenobiotic metabolism showed differential regulation throughout the estrous cycle and also during early pregnancy. The proteins associated with this pathway included sulfotransferases (SULT), SULTA1, SULTC1, COMT, PPP1R7, PRKAR1A, and GST. SULTs and COMT are enzymes responsible for metabolism of neurotransmitters and hormones such as estradiol [44]. The role of E2 in the luteal function in pigs is not clearly understood. Whereas some studies report its beneficial effect on CL function [45,46], others suggest E2 to play a role in CL regression [22,47]. Our results suggest that actions of E2 must be dependent upon the expression of E2-metabolizing enzymes, which differ in abundance during the estrous cycle. Whereas expression of SULTs such as SULT1A1 and another E2-metabolizing enzyme, COMT, was high during the mid-luteal phase, up to a ~ 3-fold decrease was observed in SULT1A1 in regressing CL as compared to functional CL. Sulfotransferases are known to regulate bioavailability of E2 and, in turn, affect the genomic effects of E2-induced gene transcription [48]. We speculated that E2 can be luteotrophic or luteolytic depending upon the expression of E2-metabolizing enzymes. A higher expression of sulfotransferases in mid-cycle CL seems to be luteoprotective. We investigated the effect of inhibition of SULT activity on luteal P4 production. Our in vitro studies using luteal explants where SULT activity was inhibited by Triclosan, a potent inhibitor of SULT activity [49], reduced P4 concentration in cell culture medium of luteal explants treated with either PGF2α or E2. The effect was more pronounced in explants treated with a combination of E2 and Triclosan. In a recent study, porcine luteal cells exposed to Triclosan alone showed an increase in P4 secretion after 48 h of culture [50]. These results are contrary to what we observed. However, our study used luteal explants instead of luteal cells and the cultures were maintained for 24 h only. Though the role of estrogen metabolites and their activity in porcine CL function and regression need to be investigated in detail, our results point towards a possibility that E2 metabolism can play a role in the CL function and regression. These findings support earlier report where 2-OHE2 and 4-OHE2, metabolites of E2, inhibited P4 production from the porcine luteal cells [47] and another study suggesting the role of E2 biosynthesis in promoting luteolysis in pigs [22]. Moreover, recently, metabolites of E2 have been suggested to be playing a role in luteal function and regression in humans [51]. 

### 3.4. Oxidation Stress

A balance between the generation of reactive oxygen species (ROS) and the expression of scavengers of ROS is important in reproductive processes such as ovulation, maintenance of luteal function, and luteal regression. A disturbance in this balance induces apoptosis and dysregulates cholesterol transport in CL [52]. Development and growth of CL from early- to mid-luteal phase was associated with the increasing abundance of proteins involved in the metabolism and scavenging of reactive oxygen species. Many of the antioxidant enzymes, GPX3, GSTA1, GSTM1, TXNDC5, and PRDX6, playing major roles in removing reactive oxygen species were identified in this study. Anti-oxidation proteins and enzymes were expressed maximally in functional CL on Day 9 of the estrous cycle followed by a decrease in most of these proteins during CL regression. Furthermore, a pregnancy-induced rescue in the expression of enzymes involved in ROS stress response was noted. The role of PRDX1 was studied in detail in mouse CL where its knockdown interfered with P4 secretion and led to luteal deficiency [53,54]. In pigs, lipid peroxidation, a source of ROS generation, occurs throughout the luteal life span [55]. Higher abundances of enzymes such as PRDX6 and GSTA1 can protect CL function against lipid peroxidation-generated oxidative stress. The other proteins belonging to anti-oxidative stress response included AKR1B1, ALDH2, COX5B, and FKBP5. Aldo-keto reductases (AKR1B1) are also induced in the response to oxidative stress and detoxify carbonyl compounds resulting from oxidation of fatty acids or lipids during CL function [56].

The importance of antioxidant enzymes in the control of CL function and regression has been shown in many studies including in rats [57], mice [58], women [59], and sheep [24]. In our study, besides all the known antioxidation enzymes belonging to glutathione peroxidase and reductases that have been previously described in rat, sheep, and human CL, we observed a luteal stage-dependent increase in the expression of mitochondrial cytochrome C oxidase 5B (COX5B) and NADH ubiquinone complex. Cytochrome c oxidase, an enzyme in mitochondrial electron transport chain, catalyzes the reduction of oxygen to water. This enzyme has not been reported in porcine CL before. During steroid biosynthesis, mitochondria are active and play a role in the conversion of cholesterol into progesterone. Functionally active mitochondria are the sites of oxygen radical production and release ROS. Besides mitochondrial GSTs, COX5B plays an important role in alleviating mitochondrial ROS. An increase in COX5B and NDUFS3 can maintain mitochondrial biogenesis and function against oxidative stress during luteal development and function [60]. The importance of this enzyme is further highlighted by its decrease during luteal regression and pregnancy, and it was observed to rescue the luteal expression of COX5B. Transition to the regression phase of the estrous cycle was associated with a decrease in the expression of antioxidative enzymes. These findings suggest that the CL function is supported by a higher abundance of components of the antioxidant system during estrous cycle and pregnancy.

### 3.5. Cellular Homeostasis and Apoptosis

Proteins associated with outgrowth of the cell, cell survival, and homeostasis and inhibition of apoptosis were found at higher abundances during Days 9–12 of the estrous cycle. The apoptotic pathway is a major regulator of CL function and regression. Survival or apoptosis of the luteal cells is precisely controlled by interactions between survival and apoptosis pathways. The mid-luteal phase of the estrous cycle is associated with high steroidogenic activity, resulting in increased metabolism. This situation gives rise to the oxidation stress and apoptosis. Therefore, a balance between pathways associated with cell survival and apoptosis is extremely necessary for the CL to be functional. Though we did not detect general modifiers of apoptosis regulation, many proteins detected in our study in mid-luteal CL are known to have a pro-survival role. Besides proteins alleviating oxidative damage to induce cellular homeostasis, proteins that can induce cell survival and inhibit apoptosis such as DPP3 and its interacting partners HSPA8, ATPase H+ transporting subunit, and NDUFS were found to be at higher abundances in the mid-luteal phase on Days 9 and 12. An increased abundance of protein DPP3 in functional CL can be associated with oxidative stress-induced apoptosis [61]. DPP3 is an anti-apoptotic protein that functions in cell survival, cell migration, and apoptosis [62]. It has been suggested that DPP3 expression can repress caspase8 and caspase3 activation and protect cells from apoptosis [62]. Dipeptidyl peptidase IV has been previously reported in human CL where it was suggested to be involved in luteal function [63]. Another protein that was in high abundance during the mid-luteal phase was HMGB1, a negative regulator of apoptosis that promotes cell survival and STOML2, a pro-survival protein that also promotes biogenesis of mitochondria. HMGB1 expression has been reported in human endometrium recently, where it not only promoted cell proliferation but was also reported to preserve progesterone receptor signaling [64]. Additionally, HMGB1 is an inhibitor of macrophage accumulation and activity [64,65], an important source of luteolytic TNFα in pigs [66].

### 3.6. Angiogenesis

A hallmark of CL growth and steroidogenesis across species is the extensive development of vasculature and angiogenesis that involves formation of new capillary blood vessels from preexisting ones. The formation of new vessels is important for transporting cholesterol to the luteal cells for P4 production. Whereas the angiogenetic pathway was enriched during the development of CL, a significant decrease in proteins associated with it was observed during the late luteal phase when CL undergoes regression. Angiogenesis in porcine CL is a well-investigated process and the role of many proteins such as FGF2, ANGPT1, and HIF has been established [10]. The importance of FGF2 in luteal angiogenesis was shown in bovine CL, where inhibiting FGF2 action led to a decrease in angiogenesis and P4 production [67]. In our study, the development of CL from Day 3 to Day 9 was associated with a significant decrease in the abundance of PTX3, an antagonist of FGF2 function. The function of PTX3 has not been reported in porcine CL; but, in CL of cows, PTX3 was one of the genes induced by PGF2α and it was shown to inhibit FGF2 actions in luteal endothelial cells and induce luteolysis [68]. Other proteins that are known inducers of angiogenesis and showed differential abundances included SERPINC1, RNH1, ANXA2, and CLIC4. SERPINC1 belongs to the family of serine proteases; the expression of SERPINC1 and its interacting partners FGG, FGB, and FGA was down regulated on Day 9 of the estrous cycle, which might contribute to luteal cell survival through promoting angiogenesis. SERPINC1 has been reported to be downregulated in bovine CL, where it was associated with the pregnancy-induced rescue of luteal function [69]. The role of other angiogenesis regulatory proteins, RNH1, CLIC4 and ANXA2, has not been investigated in the corpus luteum but is known to regulate angiogenesis in various tumors [70,71] or is involved in endothelial cell function [72,73]. Investigating the function of these angiogenic proteins in porcine CL can lead to new insights into mechanisms of angiogenesis in CL.

## 4. Materials and Methods

### 4.1. Animals

All procedures involving the use of animals were approved by the Animal Ethics Committee, University of Warmia and Mazury in Olsztyn, Poland, and were conducted in accordance with the national guidelines for agricultural animal care. Estrous induction and synchronization were achieved in 20 crossbred gilts (*Sus scrofa* domesticus) weighing ~100 kg. Hormonal treatment consisted of an intramuscular injection of 750 IU of equine chorionic gonadotropin (eCG; (500 IU-j.m., Syncrostim, Ceva Santé Animale, Libourne, France) and subsequently 500 IU of human chorionic gonadotropin (hCG; 500 IU Chorulon, Intervet International Boxmeer, Nederland) given 72 h later. The gilts were slaughtered at a local abattoir either on Day 3 (*n* = 4), Day 9 (*n* = 4), Day 12 (*n* = 4), or Day 15 (*n* = 4) of the estrous cycle, and Day 15 (*n* = 4) of the pregnancy. The day of pregnancy was verified by flushing out conceptuses from uterine horns using sterile phosphate-buffered saline (PBS buffer, 137 mM NaCl, 27 mM KCl, 10 mM Na_2_HPO_4_, and 2 mM KH_2_PO_4_; pH 7.4).

### 4.2. Blood and Corpus Luteum Collection

Blood was collected in ice-cold EDTA activator tubes from all gilts just before slaughter by jugular semi-puncture. Afterwards, blood samples were centrifuged at 3000× *g* for 10 min at 4 °C to collect blood plasma. Both ovaries with corpus luteum were placed in ice-cold PBS buffer, containing 100 IU of penicillin (Sigma-Aldrich, Saint Louis, MO, USA) and 100 μg/mL of streptomycin (Sigma-Aldrich), and it was transported to the laboratory. Corpora lutea were cut out from the ovaries and a part was snap-frozen in liquid nitrogen and stored at −80 °C for protein analysis, and another part was fixed in 4% paraformaldehyde for immunofluorescence staining (IF). Corpora lutea collected on Days 9 and 12 of the estrous cycle were also cut into small slices (25 mg) for in vitro experiments. The stage of the estrous cycle was confirmed by determining plasma P4 concentration.

### 4.3. In Vitro Study

Explants of corpus luteum were washed in PBS containing 100 IU of penicillin and 100 μg/mL of streptomycin. Afterwards, explants were placed in 24-well plates and pre-incubated in 1 mL of phenol red free culture medium (M-199, Sigma Aldrich, Saint Louis, MO, USA) with 5% FBS, 1% BSA and 100 IU/mL of penicillin, and 100 μg/mL of streptomycin for 1.5 h. Subsequently, explants were incubated in medium (M-199 + 1%BSA + antibiotics) with PGF2α (1 µM; Sigma-Aldrich), E2 (10 nM; Sigma-Aldrich), Triclosan (10 nM, 100nM, 1µM and 10 µM; Sigma-Aldrich), and a combination of PGF2α + Triclosan, E2 + Triclosan, and PGF2α + E2 + Triclosan for 24 h at 37 °C in a humidified 5% CO_2_ atmosphere. Doses of PGF2α and E2 were taken from the literature [10]. After incubation, medium was collected and frozen at −20 °C until assayed for hormone concentration. Corpus luteum explant viability was determined by lactate dehydrogenase (LDH) activity in the culture medium, measured spectrophotometrically at 340 nm utilizing the principle of Wartburg’s optical test [74].

### 4.4. Steroid Hormone Assays

Steroid hormone concentration in blood plasma and culture medium were determined using radioimmunoassay (DIA) kits: P4 (DIASource, Louvain-le-Neuve, Brussels, Belgium), according to the manufacturer’s instructions. Assay sensitivity was 0.05 ng/mL for P4, and intra-assay coefficients of variation were 8.3%.

### 4.5. Protein Extraction

Total protein was extracted from frozen CL tissue using a ceramic mortar and pestle, precooled with liquid nitrogen for at least 1 min. Homogenized frozen tissue was directly transferred into a lysis buffer (30 mM Tris-HCL; 7M urea; 2M thiourea; 4% *w*/*v* CHAPS; protease inhibitor). Lysates were sonicated on ice with 10 cycles of 30-s sonication followed by 30 s of cooling time in a Sonics Vibra-Cell VCX 120. The homogenates were then centrifuged at 16,000 *g* for 30 min at 4 °C and stored at −80 °C until analysis. Protein concentrations were determined using Quick Start, Bradford Protein assay commercial kit (Bio-Rad, Hercules, CA, USA) with bovine serum albumin as a standard.

### 4.6. Two-Dimensional Gel Electrophoresis (2DE)

Samples containing 600 µg of protein were resuspended in the rehydration buffer (7M urea; 2M thiourea; 2% *w*/*v* CHAPS; 10 mM DTT; 1% *v*/*v* IPG buffer pH 4–7; 0.002% bromophenol blue) to reach a final volume of 340 µL. The protein samples were loaded on 18-cm Immobiline DryStrips, pH 4–7 (GE Healthcare, Uppsala, Sweden), and rehydrated for 10 h (passive rehydration). Isoelectric focusing was performed with an IPGphor isoelectric focusing unit (GE Healthcare), and SDS-PAGE was run using the PROTEAN II XL Cell (Bio-Rad, Hercules, CA, USA) as described previously by Likszo et al. [23]. After electrophoresis, gels were fixed in methanol:acetic-acid:water (40:10:50) for 1 h and stained using a Coomassie Brilliant Blue G250 (Sigma Aldrich, Saint Louis, MO, USA).

### 4.7. Image and Data Analyses

The obtained gels were scanned with an ImageScanner III (GE Healthcare). Next, images were analyzed with the ImageMaster 2-D Platinum software version 7 (GE Healthcare) to identify spot intensities. To compare the protein spots between study groups, more than 10 spots in all gels were landmarked and normalized. The comparisons were made between different Days of the estrous cycle (DC): (1) 3 DC and 9 DC, (2) 9 DC and 12 DC, (3) 12 DC and 15 DC, and between (4) 15 DC and Day 15 of pregnancy (DP). Protein spots with a *p*-value < 0.05 by one-way ANOVA analysis, which showed an increase or decrease in relative intensity (>1.8-fold), were considered to be differentially abundant proteins. Only spots that were successfully matched on 3/4 of gel images were considered for further analysis.

### 4.8. Excision of 2D-Gel Spots, Tryptic Digestion, and MALDI-TOF/TOF Analysis

Spots that showed significant differences in a specific comparison were manually excised from the gel and prepared for identification using a MALDI-TOF tandem mass spectrometer (Autoflex Speed, Bruker Daltonics), as previously described by Likszo et al [23]. Briefly, protein spots were excised and digested using an in-gel tryptic digestion kit (Thermo Fisher Scientific, Waltham, MA, USA), according to the manufacturer’s instructions. Next, the peptides were desalted with C18 zip tips (Sigma Aldrich) and suspended in 2 µL of 50% acetonitrile in 0.1 trifluoroacetic acid. One μL of a peptide-matrix mixture was spotted on the MALDI target plate and left to dry at room temperature. The digested peptides were analyzed with a MALDI-MS/MS mass spectrometer, Autoflex-TOF/TOF (Bruker Daltonics, Bremen, Germany), in positive ion reflector mode with an accelerating potential at 20 kV with eight shots per second [23]. Collected MS and MS/MS LIFT spectra of selected ions were internally calibrated using monoisotopic [M + H]^+^ ion peptide calibration standards (Bruker Daltonics) and imported to BioTools (Bruker Daltonics). Peptide mass finger printing (PMF) and fragment mass spectra (MS/MS) for each individual spot were combined and used to search against the National Center for Biotechnology Information *Sus scrofa* protein database (searched on 7 January 2020) using the MASCOT Server (Matrix Science, London, UK) with the following settings: cleavage enzyme, trypsin; max missed cleavages, 2; fragment ion mass tolerance, 0.5 Da; parent ion mass tolerance, 200 ppm; alkylation of cysteine by carbamidomethylation as a fixed modification; oxidation of methionine as a variable modification. The searched results were filtered with a significant threshold of *p* < 0.05 and a Mascot ion score cutoff of ≥30 for at least two peptides.

### 4.9. In Silico Functional Analysis

Core analysis of protein was implemented by Ingenuity^®^ Pathways Analysis (IPA, Ingenuity Systems, Mountain View, CA, USA), where proteins are analyzed using canonical pathways and biological function. IPA identifies networks of interacting proteins and connects identified proteins in the data set to molecular networks contained within the Ingenuity Knowledge Database. Fisher’s exact test was used to calculate a *p*-value for each network and a functional pathway to determine which pathways are significantly linked to input data that is mapped to proteins in the whole Ingenuity Pathways Knowledgebase. The right-tailed Fisher’s exact test, using a threshold of *p* < 0.05 after application of the Benjamini–Hochberg method for multiple testing correction and z-score (in case of proteins with significantly altered abundances) were used as two statistical measures for identifying significant biofunctions and upstream regulators. Functions with a positive Z-score (Z-score ≥ 2.0) value indicated its significant enrichment and a negative Z-score (Z-score ≤ −2.0) value indicated inhibition of a function for each of the analyses. 

ToppCluster software [75] was used to identify GOs enriched by differentially abundant proteins identified in corpus luteum in different stages of the estrous cycle (growth, maintenance, and regression) or early pregnancy. Results were summarized in a tabular format and visualized as a relationship network using Cytoscape 3.8.2. [76].

### 4.10. Western Blot

The 2D or 1D Western immunoblot analysis was used to validate the results obtained in the proteomics study. For the 2D Western blot, luteal protein lysate (125 µg) obtained from various Days of the estrous cycle and pregnancy (*n* = 3 each) were resolved with 7-cm IPG strips (GE Healthcare), pH 4–7 IEF. IPG strips were equilibrated as described earlier [22]. Next, strips were loaded onto a SDS-PAGE gel for 2D electrophoresis. For the 1D Western blot, protein lysates (25 µg) from CL samples were dissolved in SDS gel-loading buffer (250 mM/L Tris–HCl, pH 6.8; 10% β-mercaptoethanol; 125 mM SDS; 40% glycerol; and 0.578 mM bromophenol blue), denatured at 95 °C for 4 min, and separated on a SDS-PAGE gel. Separated proteins were transferred onto polyvinylidene difluoride (PVDF) membrane (Sigma-Aldrich) at 60 V for 90 min. Blotted membranes were washed in Tris-buffered saline (TBS-T with 0.1% Tween 20) and blocked in 5% non-fat dried milk in TBS-T for 1.5 h at room temperature. Membranes were then immunoblotted overnight at 4 °C with polyclonal rabbit or mouse antibodies diluted in TBS-T buffer as follows: anti-APOA1, anti-CP, anti-HSPB1, anti-GC, and anti-ACTB (Table S7). Subsequently, membranes were washed three times in TBS-T and incubated with anti-rabbit or anti-mouse secondary antibodies conjugated with alkaline phosphatase (Table S7) diluted in TBS-T for 1.5 h at room temperature. Afterwards, membranes were washed three times in TBS-T and immune products were visualized by incubation in a solution of alkaline phosphate buffer (100 mM Tris–HCl pH 9.5; 100 mM NaCl; 5 mM MgCl_2_) with Nitro Blue Tetrazolium (Sigma-Aldrich) and 5-bromo-4-chloro-3-indolyl phosphate (Sigma-Aldrich) in the dark. The membranes were washed in deionized water for 1 min to stop the color reaction. The intensity of the protein bands was quantified by measuring optical density using a ChemiDoc™ Touch Imaging System (Bio-Rad). The signal was analyzed using the Image Lab version 5.2 (Bio-Rad) and normalized to a stable β-actin protein band.

### 4.11. Immunofluorescence

Immunofluorescence analysis was performed for the corpus luteum to validate the results from 2DE. The porcine CL collected from gilts were fixed in 4% paraformaldehyde, dehydrated, embedded in paraffin, cut into 5-µm slices, and transferred onto Superfrost silane-coated slides (Menzel Glaser, Braunschweig, Germany). Afterwards, the paraffin sections were incubated at 56 °C overnight, deparaffinized in xylene, and rehydrated in ethanol gradient (100%, 70%, 50%, H_2_O). Antigen retrieval was performed in citrate buffer (sodium citrate, 0.05% Tween20, pH 6.0) for 40 min at 90 °C. After cooling to RT, sections were washed three times in TBS and nonspecific binding sites were blocked using Seablocking buffer (Thermo Fisher Scientific) at RT for 1 h. For immunolabeling, sections were incubated overnight at 4 °C with rabbit anti-apolipoprotein A1 polyclonal antibody. For negative control, specimens were exposed to TBS instead of a primary antibody solution. Incubations with a primary antibody were followed by incubation for 1.5 h with secondary anti-rabbit antibody conjugated with Alexa Flour 594 (Table S7). Sections were washed three times in TBS and mounted in Mounting Medium with DAPI (Abcam, Cambridge, UK). Images were processed on a Zeiss 171 LSM800 laser-scanning microscope (Carl Zeiss, Thornwood, New York), with 20/0.4 objectives. Images were analyzed using ZEN Blue 2.5 software (Carl Zeiss).

### 4.12. Statistical Analysis

GraphPad PRISM v. 7.0 software (GraphPad Software, San Diego, CA, USA) was used to perform the statistical analysis. One-way analysis of variance (ANOVA) and Sidak’s multiple comparison test was used to determine changes of protein expression in corpus luteum. Statistical analysis of changes in protein abundance in 2D electrophoresis was performed using the ImageMaster 2-D Platinum software version 7. For the PMF and MS/MS ion search, statistically significant (*p* ≤ 0.05) matches by MASCOT were regarded as correct hits.

## 5. Conclusions

While we acknowledge that procedures such as estrus induction can probably effect molecular changes, estrus synchronization does not lead to any significant change in luteal steroidogenesis in pigs [77]. Additionally, though 2D proteomics has its limitations in identifying less abundant and low-molecular-weight proteins, our study resulted in identification of many proteins relevant to luteal function or regression. Bioinformatics analysis further led to the identification of biological pathways that are enriched during different luteal stages. Many proteins associated with cell survival, such as DPP3, HMGB1, and STOML2; estradiol metabolism such as SULTs and COMT; endocytosis such as CLTA, EZR, and SNX6; and angiogenesis, such as RNH1 and CLIC4 were reported for the first time in porcine CL to the best of our knowledge. Mitochondrial function is an important regulator of steroidogenesis and many mitochondrial proteins associated with its function and biogenesis were upregulated in functional CL during the mid-estrous cycle. On the other hand, luteal regression was associated with a decrease in the expression of proteins associated with cholesterol transport and its de novo synthesis, positive acute phase response; upregulation of peptidases such as CLPP, and decrease in resolution of oxidative stress response. Our results support the earlier suggestions that P4 has a protective effect on CL against luteolysis. Decreased abundance of proteins responsible for intake of cholesterol and changes in estrogen metabolism, followed by a decrease in anti-apoptotic protein abundances, appear to be factors contributing to porcine CL regression. The pregnancy maintains the abundances of proteins that are otherwise decreased during the late-luteal phase. An increase in the abundances of proteins associated with endocytosis, synthesis of lipids and fatty acid metabolism, inhibition of apoptosis and reactive oxygen species marks the rescue of the luteal function.

## Figures and Tables

**Table 1:** IPA analysis overview: top molecular and cellular functions, and canonical pathways associated with differentially regulated proteins between various comparisons: 3DC vs. 9DC, 9DC vs. 12DC, 12DC vs. 15DC, and 15DC vs. 15DP.

	Top Molecular and Cellular Functions	*p*-Value	No. of Molocules	Canonical Pathways	*p*-Value	No. of Molecules
3DC vs. 9DC	Cellular Compromise	5.36 × 10^4^–3.99 × 10^20^	48	Acute Phase Response Signaling	3.41 × 10^15^	18
	Post-Translation Modification	4.01 × 10^4^–2.13 × 10^17^	28	Xenobiotic Metabolism PXR Signaling Pathway	2.11 × 10^9^	13
	Cell Death and Survival	4.80 × 10^4^–9.20 × 10^17^	105	LXR/RXR Activation	3.66 × 10^9^	11
	Protein Folding	2.44 × 10^16^–2.44 × 10^16^	17	RHOA Signaling	3.54 × 10^8^	10
	Protein Synthesis	4.03 × 10^4^–9.63 × 10^16^	61	Clathrin-mediated Endocytosis Signaling	2.15 × 10^6^	10
9DC vs. 12DC	Cellular Funtion and Maintenance	4.93 × 10^3^–1.45 × 10^7^	38	NRF2-mediated Oxidative Stress Response	4.00 × 10^6^	7
	Cell Death and Survival	4.83 × 10^3^–1.83 × 10^7^	34	Akryl Hydrocarbon Receptor Signaling	5.06 × 10^6^	6
	Cell-to-Cell Signaling and Interaction	5.50 × 10^3^–3.67 × 10^7^	25	Remodeling of Epithelial Adhrerns Junctions	3.74 × 10^5^	4
	Post-Translation Modification	2.75 × 10^3^–1.80 × 10^6^	13	Glutathione-mediated Detoxification	9.35 × 10^5^	3
	Protein Folding	1.80 × 10^6^–1.80 × 10^6^	6	Xenobiotic Metabolism PXR Signaling Pathway	1.84 × 10^4^	5
12DC vs. 15DC	Cell Death and Survival	1.64 × 10^3^–3.81 × 10^11^	43	Xenobiotic Metabolism PXR Signaling Pathway	6.77 × 10^9^	9
	Cellular Funtion and Maintenance	1.68 × 10^3^–1.82 × 10^9^	28	Glutathione Redox Reactions I	1.16 × 10^8^	5
	Cellular Compromise	9.49 × 10^4^–2.57 × 10^8^	23	Remodeling of Epithelial Adhrerns Junctions	6.15 × 10^8^	6
	Cell-to-Cell Signaling and Interaction	1.39 × 10^3^–2.72 × 10^8^	26	LPS/IL-1 Mediated Inhibition of RXR Function	7.17 × 10^8^	9
	Small Molecule Biochemistry	1.24 × 10^3^–2.72 × 10^8^	27	Clathrin-mediated Endocytosis Signaling	2.06 × 10^6^	7
15DC vs. 15DP	Cell Death and Survival	7.19 × 10^3^–7.49 × 10^11^	51	Acute Phase Response Signaling	6.58 × 10^11^	11
	Lipid Metabolism	7.19 × 10^3^–1.00 × 10^7^	19	LXR/RXR Activation	1.49 × 10^8^	8
	Small Molecule Biochemistry	7.19 × 10^3^–1.00 × 10^7^	42	RHOA Signaling	3.28 × 10^7^	7
	Free Radical Scavenging	7.02 × 10^3^–1.47 × 10^6^	17	Clathrin-mediated Endocytosis Signaling	4.87 × 10^7^	8
	Protein Synthesis	2.43 × 10^3^–2.05 × 10^6^	24	Superwathwy of Choresterol Biosynthesis	3.48 × 10^6^	4

## Data Availability

All data sets generated for this study are included in the article/Supplementary Materials.

## Supplementary Materials

The following are available online at https://www.mdpi.com/article/10.3390/ijms222111740/s1.
