# Supplementary material for: Changes in Porcine Corpus Luteum Proteome Associated with Development, Maintenance, Regression, and Rescue during Estrous Cycle and Early Pregnancy
Source: Int J Mol Sci. 2021 Oct 29;22(21):11740. doi: 10.3390/ijms222111740 (PMC8583735; doi:10.3390/ijms222111740)
Supplement: Supplementary file 1 [file ijms-22-11740-s001.zip › Supplementary figures.pdf]

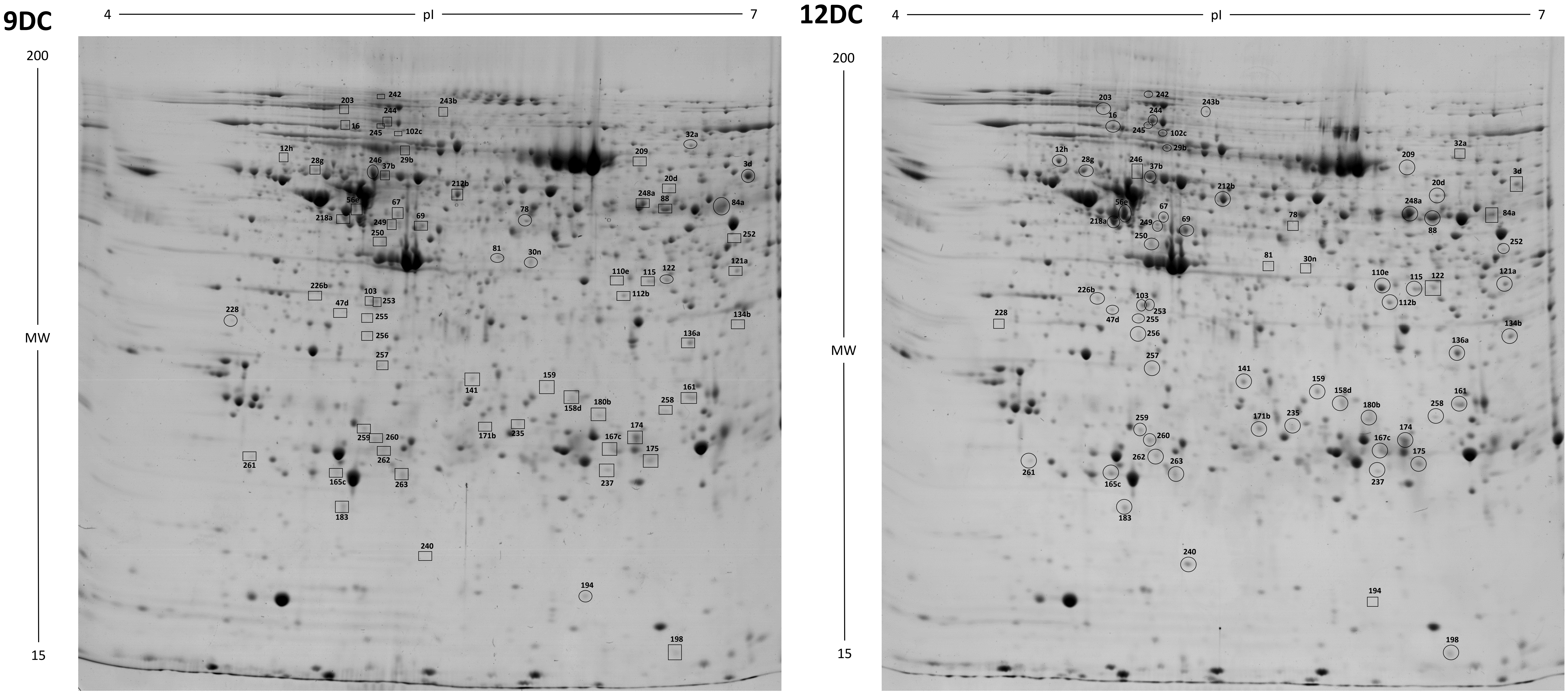

Figure S1: 2-Dimensional gel electrophoresis analysis of corpus luteum proteins. Representative 2DE gel image of differentially abundant protein spots in corpus luteum on Day 9 and Day 12 of the estrous cycle. Spots that changed significantly ($p<0.05$) between two groups are shown in the map and indicated by numbers. Proteins corresponding to spot numbers are listed in Supplementary Table S3

Line charts

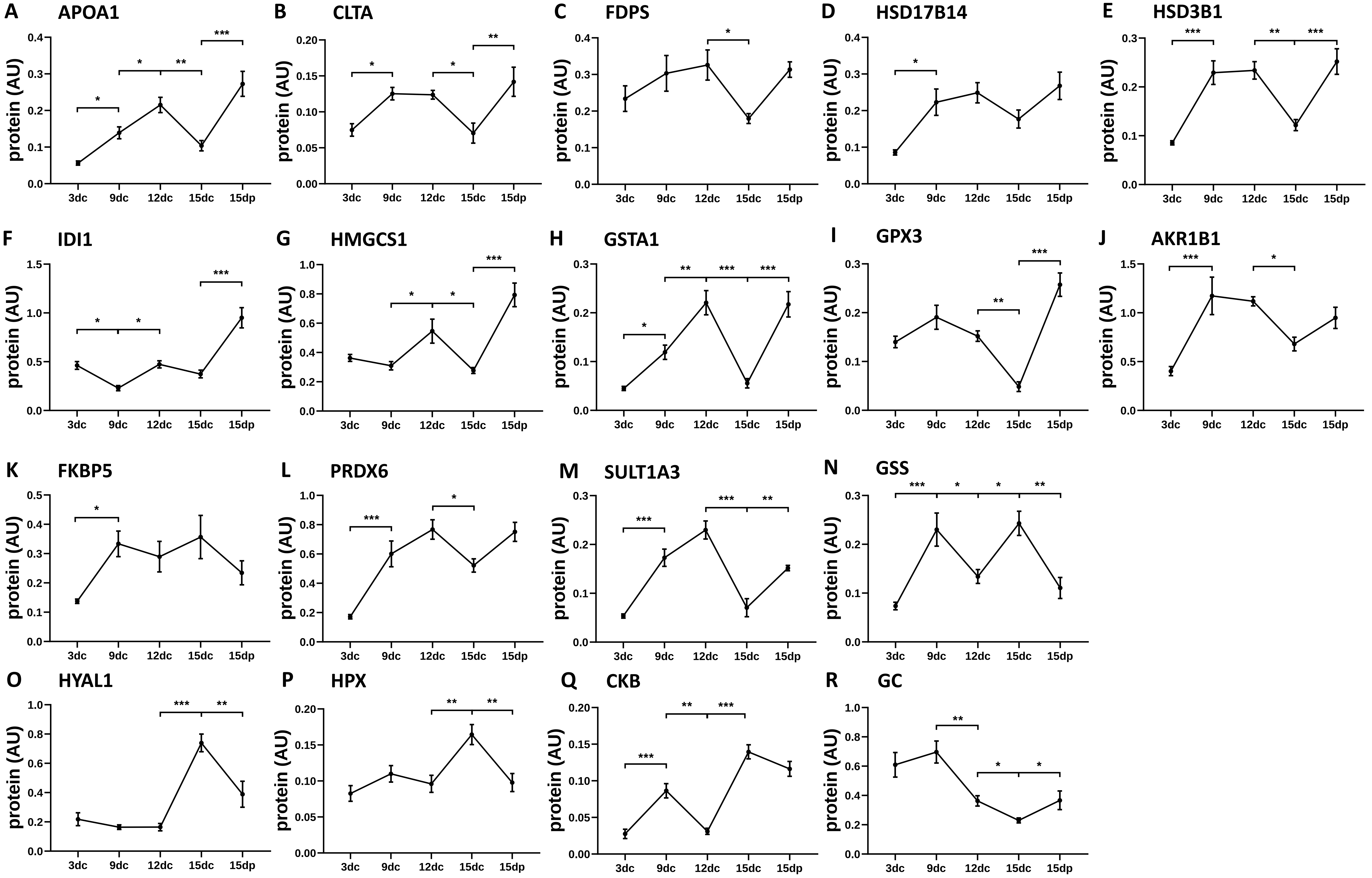

Figure S2: Changing trends in spot volumes of proteins with progression of estrous cycle from Day 3 to Day 15 And on Day 15 of the pregnancy

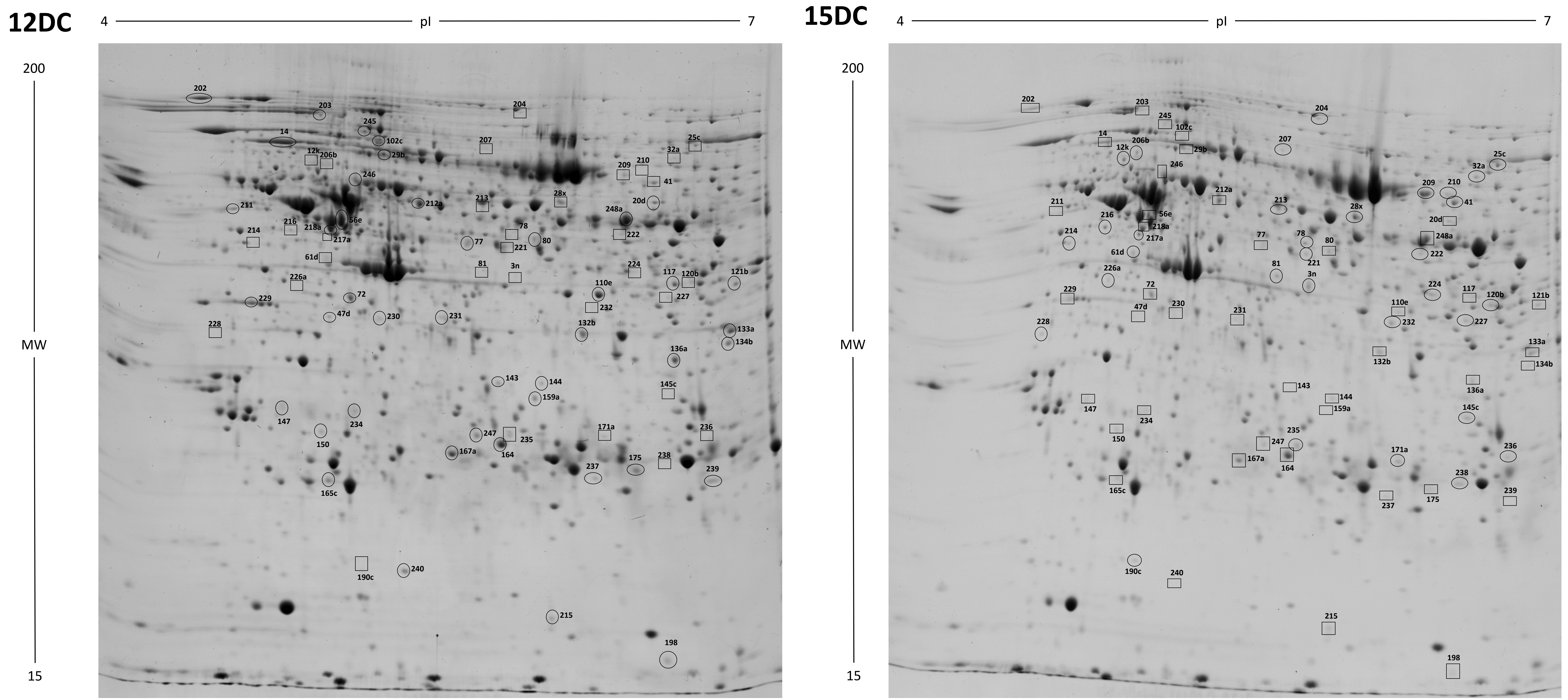

Figure S3: 2-Dimensional gel electrophoresis analysis of corpus luteum proteins. Representative 2DE gel image of differentially abundant protein spots in corpus luteum on Day 12 and Day 15 of the estrous cycle. Spots that changed significantly (p<0.05) between two groups are shown in the map and indicated by numbers. Proteins corresponding to spot numbers are listed in Supplementary Table S4

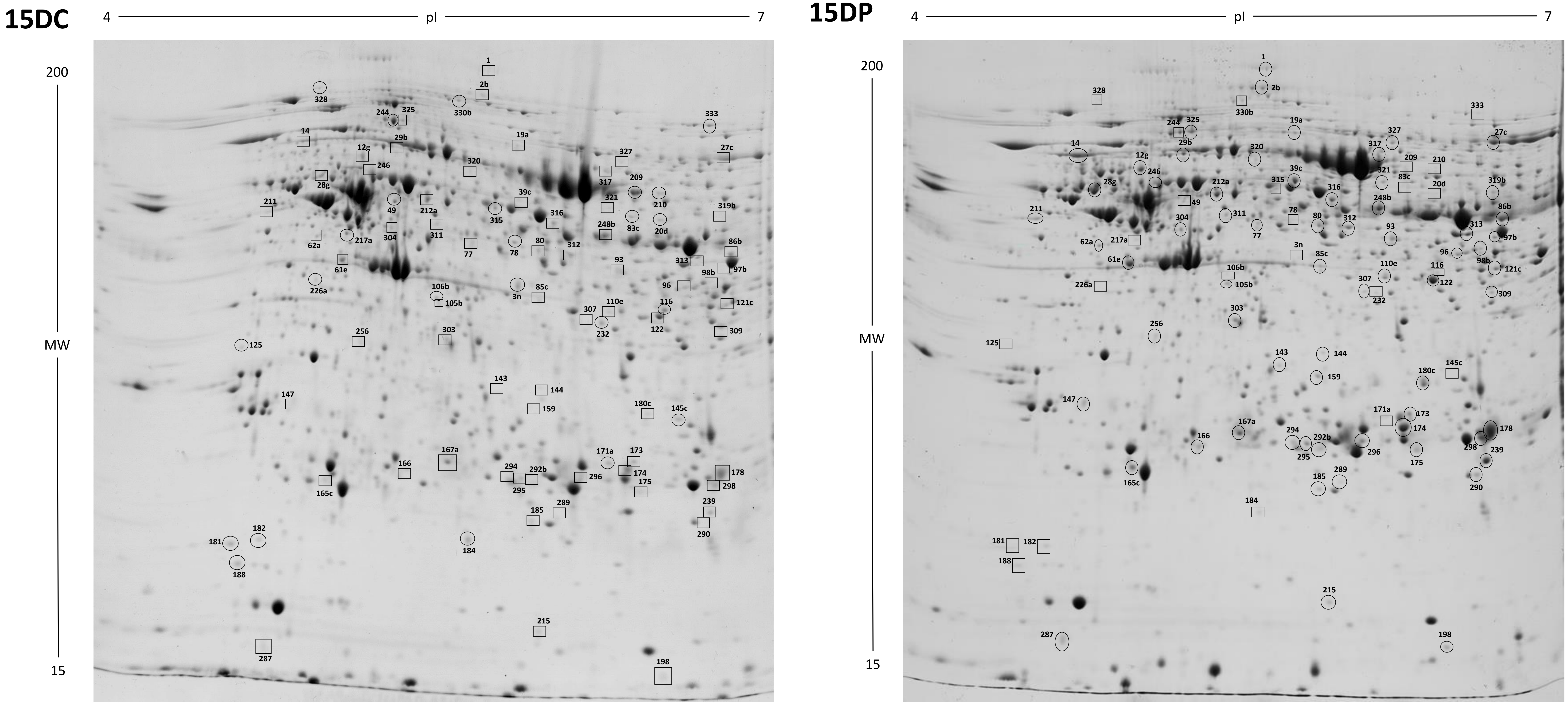

Figure S4: 2-Dimensional gel electrophoresis analysis of corpus luteum proteins. Representative 2DE gel image of differentially abundant protein spots in corpus luteum on Day 15 of the estrous cycle and Day 15 of the pregnancy. Spots that changed significantly ($p<0.05$) between two groups are shown in the map and indicated by numbers. Proteins corresponding to spot numbers are listed in Supplementary Table S5
