# Supplementary material for: Changes in Porcine Corpus Luteum Proteome Associated with Development, Maintenance, Regression, and Rescue during Estrous Cycle and Early Pregnancy
Source: Int J Mol Sci. 2021 Oct 29;22(21):11740. doi: 10.3390/ijms222111740 (PMC8583735; doi:10.3390/ijms222111740)
Supplement: Supplementary file 1 [file ijms-22-11740-s001.zip › Supplementary Table S1.pdf]

Table S1. Progesterone (P4) concentration in blood plasma in gilts on day 3, 9, 12 and 15 of estrous cycle and day 15 of pregnancy. Data are expressed as mean ± SEM.

Day	P4 (ng/mL)*
Day 3 of estrous cycle	5.38 ± 1.567[a]
Day 9 of estrous cycle	29.94 ± 10.86[bd]
Day 12 of estrous cycle	47.66 ± 16.24[b]
Day 15 of estrous cycle	20.92 ± 3.527[cd]
Day 15 of pregnancy	42.871 ± 8.58[b]

* Different letter indicate statistically significant differences, where $P < 0.01$
