# Supplementary material for: Changes in Porcine Corpus Luteum Proteome Associated with Development, Maintenance, Regression, and Rescue during Estrous Cycle and Early Pregnancy
Source: Int J Mol Sci. 2021 Oct 29;22(21):11740. doi: 10.3390/ijms222111740 (PMC8583735; doi:10.3390/ijms222111740)
Supplement: Supplementary file 1 [file ijms-22-11740-s001.zip › Supplementary Table S7.pdf]

Supplementary Table S7. List of antibodies used in study.

Peptide/Protein Target	Name of Antibody	Catalog No., Name of Source	Species Raised in Monoclonal or Polyclonal	Dilution used
**Immunofluorescence**				
APOA1	Anti-APOA1 Antibody	ab64308 Abcam	Rabbit, polyclonal	1:50
Anti-beta actin antibody	FITC Anti-beta Actin antibody [AC-15]	ab6277 Abcam	Mouse, monoclonal	1:1000
Anti-Rabbit, secondary antibodies	Donkey anti-Rabbit IgG (H+L) Highly Cross-Adsorbed Secondary Antibody, Alexa Fluor 594	A-21207 Initrogen	Donkey, polyclonal	1.500
**Western blot**				
APOA1	Anti-APOA1 Antibody	ab64308 Abcam	Rabbit, polyclonal	1:500
HSP27	Anti-HSP27 Antibody	ab2790 Abcam	Mouse, monoclonal	1:500
CP	Anti-CP Antibody	ab110449 Abcam	Rabbit, polyclonal	1:1000
GC	Anti-GC Antibody	ab65636 Abcam	Rabbit, polyclonal	1:500
ACTB	Anti-ACTB Antibody	ab8227 Bio-Rad	Rabbit, polyclonal	1:1000
Anti-rabbit, secondary antibodies	Anti-Rabbit IgG – ALP antibody produced in goat	A3812 Sigma-Aldrich	Goat, polyclonal	1:20000
Anti-mouse, secondary antibodies	Anti-Mouse IgG – ALP antibody produced in goat	A3562 Sigma-Aldrich	Goat, polyclonal	1:20000
